# Microengineered platforms for characterizing the contractile function of in vitro cardiac models

**DOI:** 10.1038/s41378-021-00344-0

**Published:** 2022-02-28

**Authors:** Wenkun Dou, Manpreet Malhi, Qili Zhao, Li Wang, Zongjie Huang, Junhui Law, Na Liu, Craig A. Simmons, Jason T. Maynes, Yu Sun

**Affiliations:** 1 Department of Mechanical and Industrial Engineering, University of Toronto, Toronto, ON M5S 3G8 Canada; 2 Program in Molecular Medicine, The Hospital for Sick Children, Toronto, ON M5G 1X8 Canada; 3 Department of Biochemistry, University of Toronto, Toronto, ON M5S 1A8 Canada; 4 Institute of Robotics and Automatic Information System and the Tianjin Key Laboratory of Intelligent Robotics, Nankai University, Tianjin, 300350 China; 5 School of Mechanical & Automotive Engineering, Qilu University of Technology (Shandong Academy of Sciences), Jinan, 250353 China; 6 School of Mechatronics Engineering and Automation, Shanghai University, Shanghai, 200444 China; 7 Institute of Biomedical Engineering, University of Toronto, Toronto, ON M5S 3G9 Canada; 8 Translational Biology & Engineering Program, Ted Rogers Centre for Heart Research, Toronto, ON M5G 1M1 Canada; 9 Department of Anesthesiology and Pain Medicine, University of Toronto, Toronto, ON M5S 1A8 Canada; 10 Department of Anesthesia and Pain Medicine, The Hospital for Sick Children, Toronto, ON M5G 1X8 Canada; 11 Department of Electrical and Computer Engineering, University of Toronto, Toronto, ON M5S 3G4 Canada; 12 Department of Computer Science, University of Toronto, Toronto, ON M5T 3A1 Canada

**Keywords:** Biosensors, Biosensors

## Abstract

Emerging heart-on-a-chip platforms are promising approaches to establish cardiac cell/tissue models in vitro for research on cardiac physiology, disease modeling and drug cardiotoxicity as well as for therapeutic discovery. Challenges still exist in obtaining the complete capability of in situ sensing to fully evaluate the complex functional properties of cardiac cell/tissue models. Changes to contractile strength (contractility) and beating regularity (rhythm) are particularly important to generate accurate, predictive models. Developing new platforms and technologies to assess the contractile functions of in vitro cardiac models is essential to provide information on cell/tissue physiologies, drug-induced inotropic responses, and the mechanisms of cardiac diseases. In this review, we discuss recent advances in biosensing platforms for the measurement of contractile functions of in vitro cardiac models, including single cardiomyocytes, 2D monolayers of cardiomyocytes, and 3D cardiac tissues. The characteristics and performance of current platforms are reviewed in terms of sensing principles, measured parameters, performance, cell sources, cell/tissue model configurations, advantages, and limitations. In addition, we highlight applications of these platforms and relevant discoveries in fundamental investigations, drug testing, and disease modeling. Furthermore, challenges and future outlooks of heart-on-a-chip platforms for in vitro measurement of cardiac functional properties are discussed.

## Introduction

Cardiac diseases remain a significant threat to human health, but the underlying pathological mechanisms are not fully understood^1^. Chronic cardiac injury and acute cardiac injury can each progressively lead to heart failure, a condition where there is sufficient loss of cardiac contractility to result in inadequate delivery of blood to the body^2^. Complications from heart failure are the most common reasons for hospitalizations in North America and the leading causes of death and disability worldwide^3–5^. If organ failure is severe enough, treatment can include the deployment of a ventricular assist device to take the place of a pumping heart chamber, typically as a bridge to heart transplant^6^. However, these mechanical devices can carry a high risk of stroke, blood clots, device malfunction or serious infection^7,8^. Available drug treatments show inadequate efficacy in reducing disease symptoms or improvements to patient morbidity or mortality, and can have significant adverse side effects^9,10^. The development of new pharmacologic treatments that can better correct underlying organ (and cellular) dysfunctions requires both cardiac models to recapitulate the physiology and pathophysiology of human heart tissues and technologies to quantify the dynamic changes in their functional properties under diseased conditions or during drug testing^11^.

In vivo animal models have long been the gold standard for evaluating cardiac therapeutics, in which the organ can be studied as a whole, and with the benefit of genetic manipulation^12–14^. However, animal models have nonhuman proteomes, with key differences in the expression of proteins involved in contractility, calcium handling, metabolism, and development^15,16^. Poor translation of animal-based assays has been widely recognized in preclinical drug evaluation (for therapeutic efficacy or cardiotoxicity), with one-third of all new pharmaceuticals failing during human clinical trials because of unexpected cardiac toxicity, which creates expensive late-phase drug attrition and potentially harms patients^17–19^. Additionally, the use of animal models has a low throughput, not allowing for the evaluation of large numbers of drug candidates. The difficulty of acquisition, limited lifespan in culture, and nonproliferation of human primary cardiomyocytes impede the establishment of human in vitro cardiac models^20^.

**Fig. 1:**
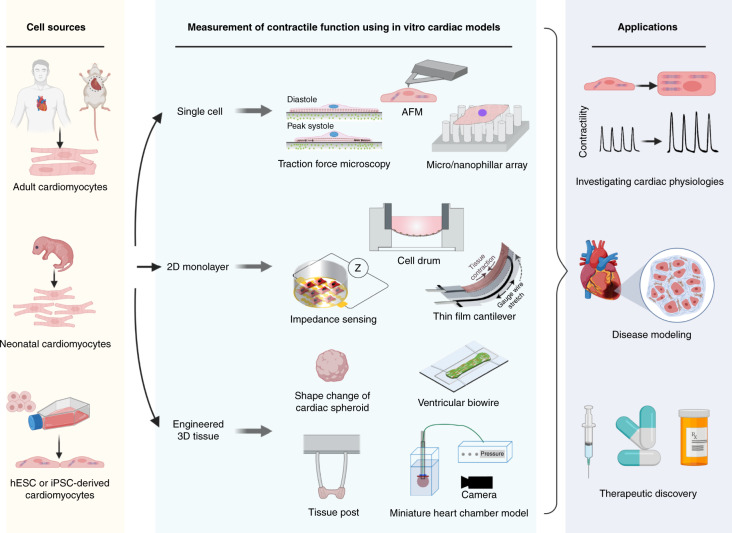
Schematic overview showing the assessment of contractile functions of in vitro cardiac models and the corresponding applications. Cell sources of in vitro cardiac models include adult cardiomyocytes, neonatal animal cardiomyocytes and cardiomyocytes derived from human embryonic stem cells (hESCs) and from induced pluripotent stem cells (iPSCs). In vitro cardiac models are established in the forms of single cardiomyocytes, 2D cell monolayers and 3D cardiac models. The assessment of contractile functions of in vitro cardiac models can facilitate research applications for fundamental cardiac physiology studies, disease modeling and therapeutic discoveries. Reproduced with permission^51,52,94,114^. Copyright 2014 the American Physiological Society, Copyright 2017 Springer Nature, Copyright 2019 Elsevier, Copyright 2015 Royal Society of Chemistry. Figure created with BioRender.com.

Due to breakthroughs in stem cell technologies^21^, cardiomyocytes derived either from human embryonic stem cells (hESC-CMs)^22,23^ or from induced pluripotent stem cells (iPSC-CMs)^24,25^ have been important preclinical study substitutes, providing reliable cell sources that contain a human proteome and avoiding the species-dependent differences present in animal models^26–28^. In addition, iPSC-CMs generated directly from patients with specific genotypes can recapitulate human disease phenotypes in vitro, facilitating the study of disease mechanisms and the development of targeted therapeutics^29^. Early in vitro cardiac models were established by culturing cardiomyocytes on standard culture dishes/plates^30,31^. However, standard culture platforms have limitations in mimicking in vivo tissue microenvironments and in evaluating cardiomyocyte functional properties^32^. Through leveraging of microfabrication technologies, emerging heart-on-a-chip platforms have been developed for culture of single cardiomyocytes^33,34^, 2D cell monolayers^35,36^, and 3D cardiac tissues^37,38^ (Fig. 1). In vitro cardiac models can be established in controlled microenvironments with integrated biosensing components for analyses of complex cell/tissue functional properties^39–42^.

Generation of a sufficient contractile force plays a central role in the pumping of oxygen-rich blood through the circulatory system by the heart^43^. At the cellular level, the contraction of cardiomyocytes follows “excitation-contraction coupling”^44^, in which the electrical action potential propagating from adjacent cells triggers membrane depolarization. The intracellular Ca^2+^ concentration then increases via Ca^2+^ influx through L-type calcium channels and intracellular calcium release from the sarcoplasmic reticulum (calcium-induced calcium release)^45,46^. Calcium ions bind to contractile proteins (troponins), initiating the relative movement of thick (myosin) and thin (actin) filaments, resulting in the shortening of sarcomeres^47^. The contractile force generated by individual cardiomyocytes is additive, which leads to synchronized beating of the human heart. The contractile functions of cardiomyocytes are typically evaluated by the following parameters: contractile force or stress, contractile strain, beating rate, beating rhythm, passive tension, active force, force-frequency relationship, force-loading relationship, synchronicity, and beating propagations^48–51^. Adverse phenotypes of cardiomyocyte contractile function, such as reduced contractility or changes to the beating rate or rhythm, are key to the pathobiology of heart disease and heart failure^5^. The contraction force of cardiomyocytes or heart tissues is an essential parameter in assessing cardiac pathophysiology and responses to pharmacological interventions. Therefore, it is necessary to integrate biosensing components on the heart-on-a-chip platform to evaluate the contractile functions of in vitro cardiac models.

Several biosensing techniques have been implemented in heart-on-a-chip platforms for the measurement of contractile functions of in vitro cardiac models^49,51–54^. Existing review papers have focused on the biological development of in vitro cardiac models^55,56^, with contractility measurement only briefly discussed. We comprehensively review the development of biosensing platforms for the measurement of contractile functions of in vitro cardiac models. We classify the platforms based on different in vitro cardiac models (single cardiomyocyte, 2D monolayer, and 3D cardiac tissue) and summarize and compare their sensing principles, measured parameters, sensing performance, contractile force/stress magnitudes, cell sources, and cell/tissue model configurations. The applications and discoveries enabled by these platforms in drug testing and disease modeling are also reviewed. We further discuss current challenges and future trends for analyses of the contractile functions of in vitro cardiac models with heart-on-a-chip platforms for investigation of cardiac physiology and disease mechanisms, evaluation of drug-induced responses, and development of regenerative therapies.

## Platforms for in vitro contraction measurement

### Contractile function measurement of single cardiomyocytes

Techniques for cardiomyocyte isolation were developed 40 years ago^57^, followed by the development of in vitro cardiac models in the configurations of single cardiomyocytes^33,34^, 2D cell monolayers^35,36^, and 3D cardiac tissues^37,38^. Single-cardiomyocyte models, the earliest and simplest in vitro models, were initially established by directly isolating cardiomyocytes from adult/neonatal rat hearts^58,59^, chick embryos^60^, pig ventricular hearts^61^ and human hearts; later, they were established by utilizing cardiac cell lines (e.g., HL-1) and hESC/iPSC-derived cardiomyocytes^62^. Experiments on single cardiomyocytes allow investigations into their cellular and subcellular structures and electrophysiology but sacrifice tissue complexity (including interactions of multiple cell types) and intercellular communication^63^. Isolated cardiomyocytes can be from different regions of a heart, including the atria, left or right ventricle, or conductive system, each with a distinct phenotype^64^. However, isolated adult cardiomyocytes have limited viability in in vitro culture, exhibiting significant loss of total cell numbers (50–70%) within 1 week of isolation, time-dependent alterations to cell morphologies, and culture-related functional changes^58,61^. Hence, isolated adult cardiomyocytes are usually used fresh within a few hours after isolation^65^. Despite these limitations, the single-cardiomyocyte model is valuable for physiological and pathological analyses of cell function, intracellular hemostasis and protein biochemistry and especially for research on adult cardiomyocytes isolated from healthy or failing human hearts^66^.

#### Two-point force transducers

**Fig. 2:**
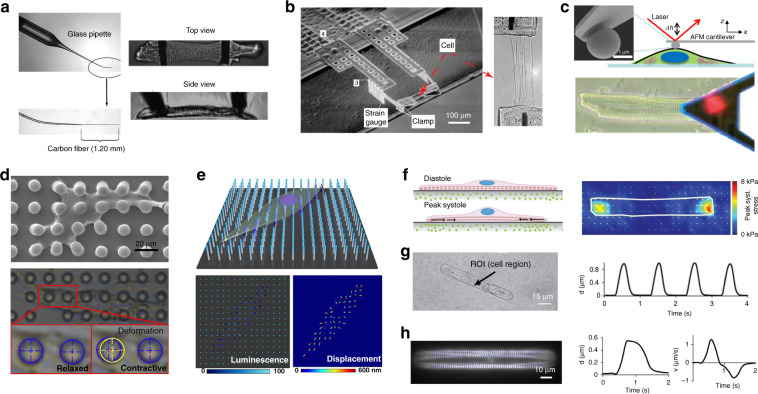
Contractile function measurement of single cardiomyocytes. **a** Carbon fiber force-length measurement system for mechanical manipulation and force measurement of intact cardiomyocytes. Cell passive/active forces are calculated from carbon fiber bending. Reproduced with permission^71^. Copyright 2007 American Physiological Society. **b** MEMS force transducer system for the contraction measurement of isolated cardiomyocytes. The right image demonstrates a single cell attached to the clamp. Reproduced with permission^76^. Copyright 2014 American Physiological Society. **c** Determination of cardiomyocyte contraction with atomic force microscopy (AFM). Reproduced with permission^219^. Copyright 2016 American Chemical Society. **d** Micropillar arrays that are 10 µm high and 10 µm in diameter. Micropillar deformation is measured to calculate cell contractility. Reproduced with permission^89^. Copyright 2018 Elsevier. **e** InGaN/GaN nanopillar arrays used for contraction measurement at a spatial resolution of 800 nm. Reproduced with permission^93^. Copyright 2021 American Association for the Advancement of Science. **f** Traction force microscopy used to measure contractile stress based on the movement of fluorescent beads embedded in a deformable gel substrate. Reproduced with permission^94^. Copyright 2014 American Physiological Society. **g** Video-based analysis of single-cell beating displacement or strain magnitude using brightfield microscopy. **h** Video-based analysis of sarcomere shortening by tracking of the movement of fluorescently labeled myofibrils (actin). **g**, **h** Reproduced with permission^99^. Copyright 2017 American Heart Association

In early apparatuses used to measure the contractile force of a single cardiomyocyte, force transducers were attached to the opposite ends of the cell. Various tools were developed to couple a single cardiomyocyte to a force transducer, including suction micropipettes^67^, adhesive-coated glass beams^68,69^, glue-coated stainless-steel needles^70^, tungsten needles^67^, and carbon fibers^71^. The contractile force was determined either through optically imaging the beam/fiber displacement^72^, monitoring bending via laser beam reflection^67^ or directly obtaining an electrical readout from the connected strain gauge^67,68^. Measurement was performed with either a skinned cell to study morphological changes (e.g., sarcomere shortening) or an intact cardiomyocyte to study the excitation-contraction coupling mechanism^73^. Among these methods, the carbon fiber technique was most commonly used since the fiber tip can naturally attach to the cell surface without the use of adhesive^71,74^. As shown in Fig. 2a, two carbon fibers were inserted into glass pipettes, mounted onto micromanipulators, and attached to the sides of the cardiomyocyte. One fiber was stiff, serving as a mechanical anchor, while the other with a compliant fiber tip that allowed for recording of force. The bending magnitude and the length of the cardiomyocyte were recorded, and the cell length and mechanical loading could be adjusted dynamically to perform force-length measurements under the physiological conditions of isometric, isotonic, auxotonic and work-loop contractions^71,75^. In addition to macrosized transducers, a miniaturized two-point force transducer only a few cubic millimeters in size was constructed using microfabrication technology^76^. As shown in Fig. 2b, cardiomyocyte contraction induced a change in the electrical resistance of the polysilicon strain gauge. The device was able to resolve forces from 100 nN to 50 µN.

#### Atomic force microscopy

Atomic force microscopy (AFM) is often used to characterize the mechanical properties of solid materials and biological samples at the micro- and nanoscopic levels^77^. AFM is capable of measuring a wide range of forces from pN to μN^78^. AFM has been applied in biology to measure cellular mechanical properties, such as elasticity, plasticity, adhesive behavior, and surface roughness^75^. The contractile force, beating rate and vertical displacement of cardiomyocytes have also been investigated by measuring the deflection of an AFM cantilever positioned on a beating cardiomyocyte^79^ (Fig. 2c shows the setup of an AFM cantilever for contractility measurement). After the cell surface is gently touched with the AFM cantilever, the z-piezo is locked in its position. Cell beating induces vertical fluctuations of the cantilever, which are detected by measuring the displacement of the reflected laser beam. The force in the vertical direction is quantified based on the deflection signals and the spring constant of the cantilever. It should be noted that AFM measurement requires direct contact with the cell, which can mechanically stimulate the cell and undesirably cause disordered beating patterns and occasional cessation of contraction^80^. Reducing the cell indentation depth to 10–30 nm and increasing cell confluency have been reported to effectively minimize the potential perturbation of cardiomyocyte beating. AFM measurements have revealed the spatial heterogeneity of contraction forces at different cell locations^81^. Hence, averaging force values from multiple cell locations can more accurately represent cardiomyocyte contractility. AFM measurement has been applied to assess contraction deficiency in diseased cardiomyocytes^80,82^ and to evaluate the effects or cytotoxicity of drugs at the single-cell level^83^. In addition, AFM has been employed to map the local elasticity^84^ of single cardiomyocytes and investigate the time-dependent relationship between contraction and intracellular calcium concentration changes^85^.

#### Micro/nanopillar array

Micropillar substrates have been used to measure traction forces generated by single cells^86,87^ including cardiomyocytes^88,89^ (Fig. 2d shows an array of flexible Polydimethylsiloxane (PDMS) micropillars with a diameter of 2–10 µm fabricated using soft lithography). After microcontact printing with an extracellular matrix (ECM) and cell seeding, cardiomyocytes adhere to and spread across the tips of multiple micropillars. The deflection of a micropillar caused by cardiomyocyte contraction is recorded and analyzed by beam-bending theory to calculate the contractile force of an individual beating cardiomyocyte^90^. Surface patterning of PDMS micropillar arrays has also been proposed to improve the structural and functional properties of cardiomyocytes. For instance, Oyunbaatar et al. integrated microgrooves into the top surface of PDMS micropillars to enhance cell alignment and therefore the contractility of the cells^91^. Mushroom-shaped PDMS micropillars with 3D topographical surfaces have also been created to increase the cardiomyocyte adhesion area^92^. Compared with those of flat micropillar surfaces, the topographical microenvironments on the mushroom-shaped micropillars improve the polymerization of actin filaments, the formation of focal adhesions and the contractility of attached cardiomyocytes. Zheng et al. recently proposed a well-aligned InGaN/GaN nanopillar array, named the piezo-phototronic light nanoantenna array, to achieve high-resolution (800 nm) mapping of force distribution across a single cardiomyocyte through dynamic changes in photoluminescence intensity or displacement at the tips of nanowires (Fig. 2e)^93^. Micropillar/nanopillar arrays can map multidirectional forces at a subcellular spatial resolution; however, the limited attachment contact areas between cells and micropillars can cause local stress concentration on cell membranes, which may influence cell morphology and physiology through mechanotransduction^73^.

#### Traction force microscopy

Traction force microscopy (TFM) is a noninvasive technique for measuring cell-level traction forces in a more native-like stiffness microenvironment^94,95^. Soft polyacrylamide gel or PDMS substrates are typically fabricated with a stiffness range of 1–30 kPa and surface-functionalized with ECM proteins for cell culture. Fluorescent beads (0.05–0.5 µm in diameter) embedded in the top surface or within the elastic substrate are used as fiducial markers^96^. Substrate deformation induced by cell beating is measured by tracking in-plane displacements of fluorescent beads between the maximum contraction and relaxation phases by image cross-correlation techniques (e.g., particle image velocimetry) (Fig. 2f)^97^. Based on the displacement field and substrate elastic modulus, the contraction magnitudes and distributions are calculated using Fourier transform traction cytometry^95^. In addition to force detection, compliant substrates can be used to implement topographical and mechanical cues to investigate microenvironmental effects on cardiomyocyte physiology. Cell shape can be controlled through micropatterning ECM proteins on the gel surface with defined aspect ratios^94^. Cardiomyocytes shaped with physiological aspect ratios (5:1–7:1) show highly aligned myofibrils along the main cell axis, as in primary adult cardiomyocytes, along with an improvement in observed cell function^41^. Through changing the substrate stiffness from healthy (22 kPa) to pathological (144 kPa), cardiomyocytes cultured on substrates with stiffness comparable to that of the native myocardium show optimal cell morphology and strong contractile force development^98^.

#### Video-based cell motion detection

Optical imaging of changes in cardiomyocyte shape or sarcomere dimensions during cardiomyocyte beating has been used as an indirect method to evaluate cell contractile performance^99^. Several kinetic parameters can be derived from videos to characterize cellular contraction, including the cell shortening ratio, beating displacement, contractile strain, contraction velocity, beating frequency, peak-to-peak time, and sarcomere shortening. After the recording of a time sequence of cardiomyocyte contraction under microscopy, image processing techniques can be utilized to quantify the cell beating behaviors, such as the periodic change in transmitted light intensity^100^, edge detection to quantify the cell shape change^101,102^, and digital image correlation to calculate contraction strain or displacement magnitude (Fig. 2g)^48,103^. Fractional sarcomere shortening can also be quantified from either brightfield videos with clear sarcomere striations or fluorescence videos of labeled sarcomeres as a metric of force production (Fig. 2h). Video-based motion analysis is a noninvasive and label-free method for contraction measurement. Advanced image processing algorithms and open-source software tools have been developed to facilitate automated measurement^99,100^. Brightfield imaging can also incorporate other techniques, such as patch clamps, MEA and voltage/calcium sensitive dyes, for multiplexed measurement of excitation-contraction coupling parameters^104,105^. Video-based motion detection has also been applied to cardiac spheroids, which have clearly defined boundaries for image segmentation. However, due to the thin, flattened geometry and high confluency, detecting mechanical motions of cardiomyocytes in a monolayer is challenging with this method. The main disadvantage of this method is the unclear relationship between kinetic parameters and contraction force. Ribeiro et al. found a difference in the magnitude of variation between traction force and beating displacement from the same cell populations in response to isoproterenol^99^. Estimation of contractility changes based on the cell’s mechanical movements may induce measurement errors.

### Contractile function measurement of 2D monolayers

Compared with single cardiomyocytes, cardiomyocytes grown as a monolayer build up critical intracellular junctions, which play key roles in action potential propagation and synchronous beating^106,107^. Conventional 2D cardiac models were established by culturing cardiomyocyte monolayers on standard culture dishes and in multiwell plates. Recent developments in microfabrication techniques have enabled the culturing of 2D monolayers on micro-physiological systems that allow for precise control of microenvironments in vitro^39,108^ and in situ measurement of cardiac functional properties^42,49^. Although 2D monolayer models have limitations in providing 3D extracellular microenvironments and in coculture of cardiomyocytes with other cell types^109^, 2D laminar cardiac models render a balance between model complexity and the maintenance of key cell physiologies for functional tests. Thus, they are commonly used in industry to test the therapeutic and cardiotoxic effects of drug candidates with high throughput^52,110^.

#### Electrical impedance-based contractility sensing

**Fig. 3:**
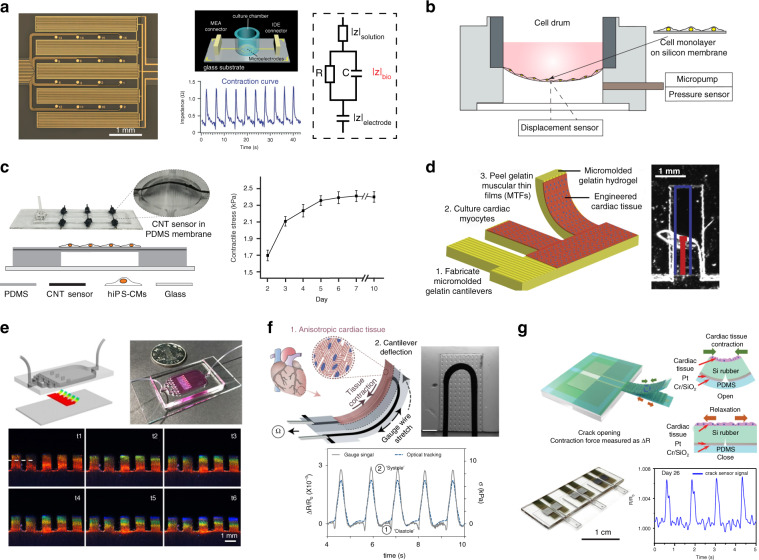
Contractile function measurement of 2D cardiomyocyte monolayers. **a** Picture and schematic of an impedance-based sensor to record contraction of cardiomyocytes grown on interdigitated electrodes. R and C in the simplified equivalent circuit model represent the resistance and capacitance of the cardiomyocyte monolayer. Beating activities are reflected by the dynamic changes in impedance signals. Reprinted with permission^108^. Copyright 2017 Royal Society of Chemistry. **b** CellDrum used to measure the contraction-dependent membrane deflection by using a laser sensor or the oscillating differential pressure change by using a pressure sensor. Replotted based on^119^. Copyright 2016 Elsevier. **c** Flexible membrane device integrated with CNT composite strain sensors for the measurement of cardiac contractility. The data plot reveals contraction development with increasing culture days. Reprinted with permission^120^. Copyright 2018 American Chemical Society. Muscular thin-film (MTF) platforms are developed by seeding anisotropic cardiomyocyte monolayers on **d** micromolded gelatin hydrogel cantilevers, **e** biohybrid structural color hydrogel cantilevers, **f** 3D-printed flexible cantilevers with embedded carbon black strain gauges, or **g** microfabricated silicone cantilevers with embedded high-sensitivity crack sensors. The contraction of cardiomyocyte monolayers is measured by **d** optically tracking the cantilever curvature change, **e** imaging the hydrogel color shift from diastole to peak systole and **f**, **g** recording the resistance change of embedded strain sensors. Reproduced with permission^52,121,124,126^. Copyright 2014 Elsevier, Copyright 2018 American Association for the Advancement of Science, Copyright 2017 Springer Nature, and Copyright 2020 Springer Nature

Electrical impedance measurement using interdigitated electrodes (IDEs) was originally developed to quantify changes in cell adhesion, morphology, proliferation, migration and confluency^111,112^. As shown in Fig. 3a, after a low AC potential is applied across the IDEs, an ion current flow is generated between the electrode pairs. The ion current changes when cells attach and spread on the electrode surface, which is measured by electrical impedance spectroscopy^113^. The equivalent circuit model of the measured impedance signal is composed of the electrode impedance, solution impedance and cell-electrode interface impedance. Cardiomyocyte morphological changes during dynamic beating induce variations in impedance magnitude. The cell index value, calculated as the relative impedance change (ΔZ/Z_0_) during cell beating, is commonly used as the metric to quantify cardiac contractility^114^. Impedance biosensors enable label-free, quantitative and long-term recording of cellular signals, including beating magnitude, beating frequency and rhythm^115^. They also have the potential to be integrated with other sensors for multiparameter measurement of cardiac functional properties. Multiwell impedance sensor plates together with multichannel recording instruments have been developed for high-throughput evaluation of drug effects and cardiotoxicity^115,116^. However, care needs to be taken in interpreting impedance data since several factors can influence the signal patterns, such as the electrode dimensions, sweeping frequency, cell adhesion and variation in cell confluency among different culture days. The external AC current flow may introduce undesired side effects and perturb normal cellular physiology.

#### Flexible thin-film platforms

The contraction of a 2D monolayer can be characterized by the mechanical deflections of a flexible thin film induced by cell beating. CellDrum is a flexible circular membrane device in which a thin silicone membrane substrate is sealed and suspended at the bottom of a cylindrical culture chamber (Fig. 3b)^117,118^. A rubber ring is used to fix the CellDrum for cell culture and contraction measurement. The flexible and thin membrane allows cell adhesion and spreading to form a cardiomyocyte monolayer in vitro. Rhythmic contraction of cardiomyocyte monolayers lifts the membrane upward. Contractility measurement is conducted by either using a pressure sensor to record the differential pressures inside the chamber or using a laser deflection sensor to record the membrane deflections. The contractile force can be calculated based on the magnitude of pressure/displacement changes and the dimension of the CellDrum (16 mm in diameter and 4 µm in thickness)^119^. Wang et al. further integrated carbon nanotube (CNT) composite strain sensors into a PDMS suspended membrane (Fig. 3c)^120^. The flexible membrane device array was used to perform continuous measurement of contractility, beating rate, and beating rhythm in the incubator environment.

2D cantilevers have also been developed for the measurement of contractility in 2D cardiomyocyte monolayers using various materials and fabrication methods. For instance, rectangular or spiral-shaped SU-8 cantilevers have been constructed using standard photolithography^35,52^. To enhance contraction-induced cantilever deflections, hydrogel or polymer materials with tunable elastic moduli have been applied to fabricate muscular thin-film cantilevers by micromolding, laser engraving or 3D printing^49,52,121^. In terms of sensing principles, surface stress generated by cardiomyocyte contraction causes mechanical bending of the flexible cantilevers, which can be measured by optical imaging, laser reflection or sensing with embedded strain sensors.

For optical imaging, dynamic deflections of the cantilevers from full contraction to relaxation are recorded via a video camera. The generated contractile stress can be either indirectly represented by cantilever displacement magnitudes^122^ or calculated using Stoney’s equation based on changes in the radius of curvature and the cantilever material properties^52,121^. To achieve accurate displacement measurement, a laser vibrometer-based measurement system has been constructed that consists of a laser vibrometer, a motorized stage, a table-top incubator and SU-8 cantilevers integrated with Au reflective plates^35,123^. A laser beam is irradiated onto the reflective plate of the cantilever, while cantilever displacement is accurately measured as laser vibration magnitudes using a photodiode. Fu et al. fabricated a free-standing hydrogel cantilever using a biohybrid color hydrogel that displays autonomic iridescence (Fig. 3e)^124^. Morphological changes in the cantilever induced by the contraction and relaxation of cardiomyocytes on the hydrogel surface are observed as the synchronous shifting of colors (from 605 to 570 nm) using an optical spectrometer. Optical tracking of the bending magnitudes of the flexible cantilevers provides an accurate way to evaluate the contractility of cardiomyocytes adhered on the cantilever surface; however, the use of a microscope or laser source requires repeated and time-consuming alignment procedures.

To measure cardiomyocyte contraction from electrical readouts, flexible strain sensors have been integrated into the cantilever platforms. Gold thin-film strain sensors have been deposited on PDMS cantilevers and insulated by a thin PDMS layer^49,125^. The contraction of a cardiomyocyte monolayer is measured as changes in the resistance (*ΔR*) of the strain sensors (gauge factor 0.58)^125^. Lind et al. introduced a multi-material 3D printing method to fabricate a multilayer cantilever with embedded carbon black composite strain sensors (Fig. 3f, gauge factor 2.56)^52^. To further improve the sensing performance, a high-sensitivity crack strain sensor has been integrated by depositing platinum on silicone film and then applying a 2% strain to the substrate for the formation of microcracks^126^. The cracked sensor-integrated silicone cantilever demonstrates a high sensitivity with a gauge factor of ~100 at a strain of 0.16% and long-term stability for continuous contractility measurement. Ultrasensitive strain sensors with precisely defined nanotrack patterns have been recently proposed to further improve the sensing performance in terms of repeatability and sensitivity^127,128^. To mimic the anisotropic structures of the native myocardium, different techniques have been utilized to create confluent, uniformly aligned cardiomyocyte monolayers. Microcontact printing is commonly used to print patterned ECM proteins onto the culture surface^121,129^. The micro-/nanogrooved top surface can also be integrated on the flexible cantilever to provide topographical cues for cell alignment^122,130^. Compared with isotropic monolayers, the improvement of cell alignment on 2D cantilever platforms has been proven to promote cell contractility, conduction velocity, and structural maturation.

### Contractile function measurement of 3D cardiac tissues

Advances in microfabrication technologies and tissue engineering have enabled the development of engineered 3D cardiac tissue models^131–133^. In vitro 3D cardiac tissues can be formed by several methods First, cells can be encapsulated into hydrogels (collagen, fibrinogen, Matrigel, and methacrylated gelatin)^132,134^. The gel matrix forms specific shapes in the culture molds during gelation and compacts into a solid tissue with time^135^. Second, cells can be cultured on the surface and in the cavities of either a natural decellularized matrix^136^ or artificial scaffolds (e.g., electrical spinning-formed fibers^137^, microfabricated polymer scaffolds^131,138^, and porous biomaterials^139^). Third, the self-assembly properties of cells can be utilized to directly form scaffold-free tissue aggregates and spheroids^140^. Compared to 2D monolayers, 3D engineered cardiac tissue can better recapitulate the normal physiological microenvironment (e.g., ECM and stiffness^141^), certain aspects of native tissue organization (e.g., cell alignment^51^, vascularization^142,143^ and interactions between multiple cell types^54^) and tissue functions (e.g., electrical coupling and contraction^144^). Studies have shown that cardiac tissues with a cell composition of 75% cardiomyocytes and 25% fibroblasts yield optimized tissue remodeling dynamics and enhanced structural and functional properties^54^. The incorporation of endothelial cells in engineered myocardial tissue also promotes neovascularization and inherent cellular function^145^.

#### Contractility measurement of 3D tissue posts, cantilevers, and wires

**Fig. 4:**
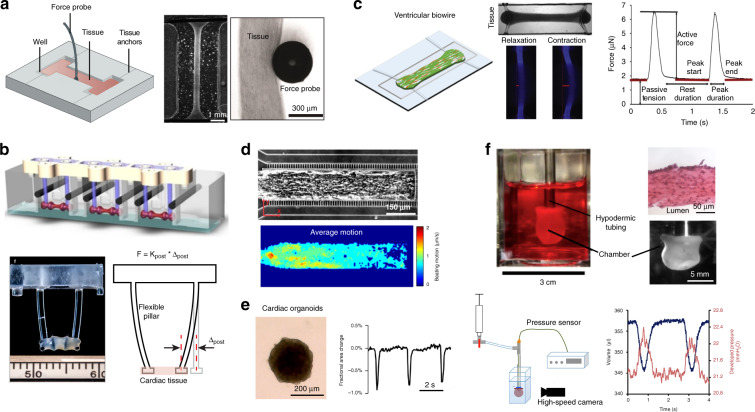
Contractile function measurement of 3D engineered heart tissues (EHTs). **a** EHT bridge across parallel rigid rods on the I-Wire platform. Tissue contraction is measured by an external force probe. Reproduced with permission^152^. Copyright 2016 Elsevier. **b** Photograph and schematic of the cardiac tissues attached at the tips of two flexible silicone posts and bending the flexible post with force. Contractile force is calculated based on the post length, stiffness and deflection. Reproduced with permission^40^. Copyright 2018 Springer Nature. **c** The Biowire II platform enables growth of thin cylindrical tissues suspended between two flexible wires that allow quantification of active force, passive tension, and contractile dynamics. Reproduced with permission^51^. Copyright 2019 Elsevier. **d** EHT developed in a confined microfluidic channel. Tissue contraction is evaluated by tracking the beating motion in the brightfield. Reproduced with permission^163^. Copyright 2015 Springer Nature. **e** Video-based contraction measurement of 3D cardiac spheroids. Contraction magnitude is represented as the fractional area change of cardiac spheroids between contraction and relaxation. Reproduced with permission^166^. Copyright 2017 Elsevier. **f** Representative images of an engineered human ventricular cardiac organoid chamber (hvCOC). Changes in the lumen pressure and volume of the hvCOC are separately recorded via a pressure catheter and high-speed video camera. Reproduced with permission^174^. Copyright 2018 Elsevier

Engineered heart tissues (EHTs) have been established by encapsulating cells (cardiomyocytes, cardiac fibroblasts, and endothelial cells) in collagen/fibrin hydrogels tethered to anchoring constructs^146^. EHTs have been widely developed for tissue physiology analysis, drug screening, disease modeling, and cardiac repair^40,51,147–150^. Early EHTs were wrapped around rigid anchoring structures such as glass/metal rods^151,152^, posts^153^, surgical sutures^154^, and steel needles^155^. The assessment of contraction was omitted^154^, conducted by inserting external probes^152^ (Fig. 4a) or conducted by transferring tissue onto a separate force measurement platform^153^. The use of external force sensors does not allow for the continuous measurement of tissue contractile functions. To solve this problem, various heart-on-a-chip platforms have been developed to integrate on-chip force-/stress-sensing components for in situ measurement of EHT contraction dynamics^132^. Cardiac tissue suspended between two flexible posts/cantilevers is a well-known model^40,132,156,157^. Cardiomyocytes are mixed with fibrin and collagen hydrogels and pipetted into casting molds in which a pair of flexible silicone posts or T-shaped vertical cantilevers are positioned (Fig. 4b). After 5–10 days of tissue compaction, cardiac muscle strips are wrapped around the silicone posts/cantilevers, exhibiting better longitudinal alignment induced by passive mechanical tension. The defection of the silicone posts or upright silicone cantilevers induced by the cyclic contraction of EHTs is video-recorded. The post/cantilever mechanical properties, bending magnitudes, and bending frequencies are used for calculation of the contractile force and beating rate of the EHT.

To improve throughput for drug testing, elastic post/cantilever platforms have been developed to construct arrays of EHTs in standard 24-well or 96-well plates^132,149^. Custom-made software has also been developed to enable image acquisition and evaluation of contractile force with increased throughput^132,149^. Based on the two-elastic post/cantilever design, other modifications have been proposed to improve platform functionalities for tissue contraction measurement. Ma et al. applied light-based 3D printing technology to fabricate a microscale force gauge made of one thin pillar as a bendable cantilever and one thick pillar as a fixed anchor^158^. Compared with the standard symmetrical design, this design simplified data acquisition and tissue contraction measurement by focusing only on the deflection magnitude of one flexible pillar. Furthermore, the bending stiffness of the flexible post was adjusted to investigate the effects of afterload (passive tension) conditions on the maturation and pathological phenotype of hiPSC-CMs^50^.

Another representative heart-on-a chip model is the flexible wire platform. Zhao et al. developed a Biowire II platform that consists of an array of microwells patterned on a polystyrene sheet by hot embossing and two flexible polymer wires located at each end of the microwell (Fig. 4c)^51,159,160^. Cylindrical trabecular tissue is formed in each microwell and suspended between two parallel polymer wires. The miniaturized microwells (5 × 1 × 0.3 mm) require fewer cardiomyocytes (~0.1 million/tissue) for tissue formation compared with other tissue models (0.5–2 million cells/tissue)^40,132,161^. Tissue contraction measurements are performed by converting wire deformation into force. The polymer wires maintain an unaltered Young’s modulus and force-displacement relationship for accurate force measurement during long-term tissue culture (up to 8 months). Compared with the two-post design, the use of elastic microwires for reliable tissue anchorage prevents potential failure of cultivated tissues due to slippage from the posts. The autofluorescence property of poly(octamethylene maleate (anhydride) citrate, POMaC) polymer wires is used for simultaneous quantification of contractile force and Ca^2+^ transients under fluorescence microscopy. In terms of drug screening, compared with PDMS, the POMaC wires show low absorption of small hydrophobic molecules, which helps improve the accuracy of dose-dependent drug screening. In addition, the generated atrial and ventricular Biowires with specifically altered gene expression exhibit chamber-specific drug responses. Another representative cardiac tissue wire model has been established that consists of a filamentous three-dimensional matrix to facilitate the adhesion and self-assembly of 3D cardiac tissues without the contribution of ECM hydrogels^162^. Synthetic parallel fibers with tunable fiber stiffness are used to anchor the 3D cardiac tissues, providing different mechanical stresses by changing the fiber bending stiffness and measuring tissue contraction through tracking of wire bending. The formation of aligned EHTs is also achieved in a confined microfluidic channel to reduce the cell cost to several thousand cells per tissue (Fig. 4d)^163^. Tissue contraction is indirectly characterized by optically tracking the beating motion under brightfield microscopy. Side perfusion channels have been constructed to recapitulate the function of the vasculature for the delivery of nutrients and drugs^164^.

#### Contractility measurement of 3D cardiac spheroids

The formation of scaffold-free 3D cardiac spheroids is based on the self-assembly property of cardiomyocytes cocultured with stromal cells or fibroblasts by using the hanging drop method^38^ or nonadhesive micromolds/substrates^165,166^. Reported spheroid diameters ranged from 200 to 400 µm, within the hypoxic limits of tissue spheroids^167,168^. The incorporation of endothelial cells helps form microvascular networks inside the cardiac spheroids and increases the permeability of oxygen and nutrients into the core region^166^. The tissue dimensions of the cardiac spheroids ensure high imaging contrast under brightfield or phase contrast microscopy for measurement of beating kinetics^100,165,169^. Similar to the video-based methods for cardiomyocyte analysis, time-series images of the spheroid shape changes induced by tissue contraction are recorded. Edge detection algorithms are applied to detect the dynamic changes in spheroid boundaries during spontaneous contraction or contraction under electrical stimulation^170^. The tissue contraction magnitude is calculated as the fractional area change of cardiac spheroids between the systolic and diastolic states (Fig. 4e)^166^. For example, Arai et al. tested the influences of mixed ratios of cardiomyocytes, fibroblasts, and endothelial cells on the formation and contraction performance of 3D cardiac spheroids. The 50:25:25 mixture composition was proven to have optimal results with regard to stable spheroid morphologies (shape and size) and apparent contractile functions^170^. However, the visible shape changes of spheroids do not directly correlate to tissue contraction force or stress. Other factors, such as cell density, tissue composition, and culture time, can influence the magnitude of size changes during spheroid contraction. In addition, nonuniform tissue sizes, cardiac spheroid rotation, and shape changes in out-of-plane directions can lead to measurement errors^171^. Nevertheless, the optical assessment of shape change can reveal drug-induced contractile responses of 3D cardiac spheroids, such as the gradual decrease in contraction amplitude upon treatment with the myosin II ATPase inhibitor blebbistatin^167^. In addition to optical imaging, Pesl et al. used AFM to measure the absolute value of contractile force together with the beating frequency of cardiac spheroids. They validated the homogeneity of contraction forces generated at different surface regions and demonstrated an increase in contractile force with increased calcium concentration^172^. A combination of MEA and AFM has been implemented to investigate the electromechanical coupling of 3D cardiac spheroids under drug testing conditions^173^. However, the simultaneously recorded extracellular field potential and mechanical contraction signals are acquired from different local regions of the spheroids, which can introduce errors in calculation of electromechanical delay.

#### Contractility measurement of miniature heart chamber models

To mimic the three-dimensional architecture and pumping functions of the heart chamber, in vitro cardiac chamber models have been established^136,174,175^. Pressure- and volume-based metrics are measured to represent the contracting or pumping performance of the engineered heart chamber, correlating to organ physiology. Ott et al. generated a bioartificial heart by reseeding rat cardiomyocytes and endothelial cells on the decellularized ECM of rat hearts^136^. Contraction of the decellularized whole heart was measured as the left ventricular pressure. By day 8 after cell seeding, the constructs showed pumping function with a magnitude of ~2.4 mmHg, equivalent to 25% of that of the 16-week fetal rat heart^176^. However, recellularization of animal cadaveric hearts with animal cell sources has impeded studies on human drug responses and prevented potential implantation into patients.

Li et al. created a functional miniature human ventricular-like cardiac organoid chamber (hvCOC) from human pluripotent stem cell-derived ventricular cardiomyocytes^174^, as shown in Fig. 4f. A hollow chamber with a wall thickness of 100 µm was formed by adding cardiomyocyte-embedded hydrogel to a mold consisting of an agarose outer boundary and a balloon core. The oscillatory pressure generated inside the lumen and cyclic changes in chamber volume were acquired simultaneously. Physiological characteristics of native ventricles, such as the developed pressure (1.26 ± 0.12 mmH_2_O), stroke volume (4.82 ± 0.63 µl), ejection fraction (2.44 ± 0.27%) and cardiac output (212.9 ± 28.6 µl/min), were measured to evaluate contractile functions. In addition, the Frank-Starling relationship was observed as a positive linear correlation between the developed pressure and loading pressure. The hvCOC represented the first engineered human cardiac construct that allowed direct evaluation of cardiac pumping performance, which previously could be monitored only in a whole heart. In addition, compared with 2D and other 3D models, hvCOCs show upregulation of the expression of proteins involved in Ca^2+^ handling, ion channels, and cardiac-specific proteins and augmented sensitivity to pharmacological intervention. It needs to be noted that the isotropic cardiomyocyte distribution in collagen-based hydrogels still shows limitations in recapitulating the organized laminar architecture of the native myocardium^177^. In the native heart, the fibrillar and anisotropic structures of the ECM provide topographical cues to guide the alignment of myocardial fibers in helical orientations^178^.

Inspired by the architecture of myocardial ECM, MacQueen et al. developed an engineered ventricle chamber model by applying ventricle-shaped nanofibrous scaffolds with sufficient porosity to guide cell adhesion and the anisotropic assembly of cardiac tissue^175^. Catheter sensors were introduced to measure the time-dependent intraventricular pressure (~50 μmHg) and volume (0.5–1 µl) produced by in vivo-like chamber contraction. A structural arrhythmia disease model was further created by adding controlled hole injuries into the tissue-engineered heart chambers. Spiral calcium waves were observed to anchor to defect locations, proving the feasibility of using the model to study arrhythmogenic heart diseases. Although current ventricle chamber models can generate elliptical pressure-volume loops similar to those of rat or human ventricles, the generated heart chamber contraction magnitudes (differences in chamber pressure), chamber volume, and corresponding ejection fractions are smaller than those of healthy mammalian ventricles by factors of ~50–250^179^. Decreasing the elastic modulus of the scaffold support, optimizing the seeded cell density, and improving tissue maturation are expected to improve the pump performance of in vitro tissue-engineered ventricle models.

## Applications of cardiac models and contractility measurement

**Fig. 5:**
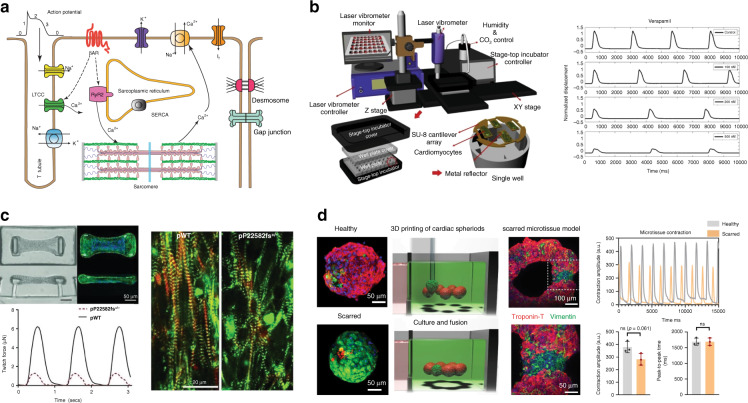
Platform applications in drug testing and disease modeling. **a** Schematic diagram showing physiological mechanisms related to cardiomyocyte contraction, including propagation of action potentials, calcium dynamics, sliding of sarcomere filaments, and intercellular communication. **b** Sample of a high-throughput platform for drug screening based on SU-cantilever arrays. Reproduced with permission^183^, Copyright 2019 Elsevier. **c** Inherited cardiac tissue model with titin-truncating variants (TTNtvs) established on a flexible microcantilever platform. The representative force curves indicate the diminished contractile performance of patient-derived (pP22582fs^+/−^) iPS-CM tissues. Compared with wild-type (pWT) cells, pP22582fs^+/−^ iPS-CMs have fewer myofibrils and abnormal sarcomeres. Reproduced with permission^190^. Copyright 2015 American Association for the Advancement of Science. **d** Acquired cardiac fibrosis model with a local scarred region established by 3D printing and self-fusion of healthy and scarred cardiac spheroids in the support hydrogel. The ratios of iPSC-CMs and fibroblasts are 4:1 for healthy and 1:4 for scarred cardiac spheroids. Reduced contraction amplitudes are observed for scarred microtissues compared to healthy controls. Reproduced with permission^200^, Copyright 2021 Springer Nature

Significant advances in the development of in vitro cardiac models and biosensing technologies for assessment of contractile functions have been made for cardiac physiology investigations, cardiac disease modeling, and therapeutic discovery. As shown in Fig. 5a, cardiomyocyte contraction is generated by a number of events, including the initiation and propagation of the action potential regulated by ion flux through various channels^44^, calcium-induced calcium release related to calcium ion channels and the sarcoplasmic reticulum^45^, sliding between sarcomere protein filaments^47^, intracellular communication^106^ and cells’ interactions with the microenvironment^180^. These complex physiological processes need to be quantified to increase the accuracy of the in vitro model, as it relates to the prediction of in vivo organ function.

### Evaluation of drug efficacy and cardiotoxicity

Evaluation of potential new therapies for cardiac diseases and preclinical safety assessment of pharmaceuticals to reduce off-target cardiotoxicity are essential for drug discovery^4^. Video-based beating analysis^4^, AFM^81^, impedance measurement^181^, flexible muscle membrane studies^49^, engineered cardiac tissue modeling^132^ and other methods described in the previous sections^71,163,166,174^ have been used to measure functional changes under different drug treatments. Candidate compounds are compared to drugs with known cardiotropic effects as part of platform validation (summarized in Table 1). Compound classes that are important for the corroboration of platform predictive ability include β-adrenergic agonists (e.g., isoproterenol, epinephrine), L-type calcium channel antagonists (i.e., verapamil), sodium channel antagonists, potassium “funny channel” antagonists (i.e., ivabradine), arrhythmogens (i.e., the hERG channel antagonists cisapride and E4031) and broad multimechanism cardiotoxins (i.e., anthracyclines, protein kinase inhibitors)^182^. To incorporate current platforms into drug discovery pipelines, contraction measurement components have been integrated to improve the throughput and scalability for large-scale preclinical drug screening^36,49^. For example, the cantilever platform has been miniaturized so that 192 cantilevers can be assembled into a single well plate (Fig. 5b)^183^, and a laser-based sensor is used to perform automated contraction measurement by tracking the cantilever displacement. Impedance measurement based on IDEs has advantages for evaluating drug efficacy and cardiotoxicity with high sensitivity, throughput and scalability. It can be used to specifically recognize ion channel blockers from electrical signals, mechanical signals and excitation-contraction coupling, providing a powerful tool for drug development^184,185^. Flexible sensors have also been incorporated into high-throughput drug-screening platforms to enable simultaneous and continuous electrical readouts of contractility changes to be obtained^52,120^. In addition to platform development, advances in iPS-CM technologies have promoted the usability of in vitro human-based models in drug development. Patient-derived iPSC-CMs have been used to simulate patient-specific drug responses for the development of precision treatment strategies^186^. To recapitulate drug effects on different heart chambers, progress in generating pure populations of chamber-specific cardiomyocyte subtypes, including atrial-like, ventricular-like and sinoatrial node-like pacemaker cells, has been made^147^. For example, the sarco-/endoplasmic reticulum calcium ATPase inhibitor thapsigargin has been reported to affect only ventricular, but not atrial, Biowire tissues due to differential sarcoplasmic Ca^2+^ handling between the atrial and ventricular myocardium^187,188^.

**Table 1:** Microengineered platforms for characterizing the contractile functions of in vitro cardiac models

Contraction sensing platform	Model configuration	Cell type	Sensing principle	Sensing performance	Contraction magnitude	Other detection parameters	Platform applications	Reference(s)
Carbon fiber-based platform	Single cell	Pig, hamster, rat and human cardiomyocytes	Contraction induces carbon fiber bending.	Sensitivity: 0.02 m N^−1^; CB fiber stiffness: 0.08–0.25 µm/µN	2.56–5.72 µN 16.2–60.9 mN/mm^2^	Passive tension, beating rate, force-length relationship (Frank-Starling’s Law), work output, instantaneous elastance	•Cell stretching •Drug testing: isoproterenol •Disease modeling: hereditary cardiomyopathy	^71,74,75,220^
MEMS force transducer	Single cell	NRVMs	Cell contraction deforms a strain gauge. The resistance change is converted to an amplified electrical output.	Resolution: 100 nN Sensitivity: 1.6 ± 0.31 mV/µN Sensing range: 100 nN–50 µN	5.77 ± 2.38 µN 14.7 ± 7.68 kPa	N.A.	N.A.	^76^
Atomic force microscopy (AFM)	Single cell/cell cluster and cardiac spheroid	NRVMs, iPSC-CMs, hESC-CMs	Cell contraction induces the deflection of the contacted AFM cantilevers in the Z direction.	Sensitivity: 60 pN Cantilever spring constant: 0.02–2 N/m Sensing range: from piconewtons to nanonewtons	0.5–25 nN	Beating rate, beating rhythm, beating duration, cellular stiffness	•Physiology: cardiomyocyte maturation •Drug testing: isoproterenol, blebbistatin, dexrazoxane, monoxime, norepinephrine, doxorubicin, metoprolol, nifedipine •Disease modeling: familial dilated cardiomyopathy, arrhythmia	^80,81,83,219,221^
Micropillar arrays	Single cell	NRVMs, iPSC-CMs	Contraction force is calculated based on micropillar stiffness and deflections.	Spring constant: 29–142 nN/µm	7.5–75 nN	Passive tension, beating rate, contraction and relaxation velocity, peak contraction power	•Physiology: influence of substrate stiffness on twitch force; influence of ECM protein on cell attachment Drug testing: verapamil, norepinephrine, doxorubicin, metoprolol, cytochalasin-D	^53,88–90^
Traction force microscopy	Single cell/cell monolayer	NRVMs, iPSC-CMs	Cardiomyocyte contraction induces movement of fluorescent beads embedded in a deformable gel substrate. Contractile stress is calculated based on the bead displacement field and gel stiffness.	Substrate stiffness: 3–144 kPa	0.05–0.34 µN 0.26–2.37 mN/mm^2^	Beating rate, contraction velocity, time to peak systole, peak systolic work	•Physiology: cardiomyocyte maturation; matrix stiffness-regulated cardiomyocyte contractility; shape-dependent cardiomyocyte contraction •Drug testing: caffeine, verapamil, isoproterenol •Disease modeling: dilated cardiomyopathy	^42,98,216,222,223^
Video-based motion detection	Single cell/cell cluster and cardiac spheroid	NRVMs, iPSC-CMs	Beating displacement, shape changes and contractile strain can be optically analyzed by edge detection or digital image correlation.	Recording rates: >70 frames per second	N.A. (indirect method)	Beating displacement, beating strain, contraction velocity, beating rate, beating duration, sarcomere shortening. time-to-peak, relaxation time	•Drug testing: isoproterenol, norepinephrine, omecamtiv mecarbil (OM), cisapride, E-4031, verapamil, quinidine, sotalol, nifedipine, aspirin, caffeine •Disease modeling: cardiac hypertrophy (decreased expression of MYBPC3)	^99,100,102,209^
Impedance sensing	Monolayer	NRVMs, iPSC-CMs, mouse ESC-CMs	Contraction induces impedance changes at the cell-electrode interface.	Sweeping frequency: 5 k–200 kHz Oscillation voltage: 10–20 mv	N.A. (Indirect method)	Beating rate, beating rhythm	•Drug testing: verapamil, blebbistatin, norepinephrine, flecainide, E-4031, carbachol, amlodipine, mibefradil, zatebradine •Disease modeling: arrythmia	^108,181,224^
CellDrum	Monolayer	NRVMs, iPSC-CMs	Synchronous beating of monolayer cells induces membrane deflection.	Sensitivity (ΔR/R_0_): 0.01 kPa^−1^	1.15–43.1 kPa	Beating rate, beating rhythm	•Drug testing: veratridine, S-Bay K8644, isoproterenol, verapamil, omecamtiv mecarbil (OM), ivabradine, E-4031	^118–120^
Thin-film cantilever	Monolayer	NRVMs, iPSC-CMs	The contraction of the cell monolayer deflects the flexible cantilever. Cantilever deflection can be measured by an optical imaging system, laser vibrometer or embedded strain sensors.	Laser resolution: 15 pm Gauge factor of strain sensors: 0.58–100	1–15 kPa	Bending displacement, beating rate, beating rhythm	•Physiology: cardiomyocyte maturation •Drug testing: verapamil, isoproterenol, quinidine, lidocaine, E-4031 •Disease modeling: Barth syndrome during ischemia–reperfusion injury	^52,123,126,191,194^
EHTs attached to rigid anchoring structures	3D tissue	NRVMs, iPSC-CMs	An external force transducer is used.	Depends on sensor specifications	0.3–0.5 mN	Passive tension, force-length relationship	•Drug testing: blebbistatin, isoproterenol, epinephrine	^225^
Tissue posts and cantilevers	3D tissue	NRVMs, iPSC-CMs	Cantilever/post deflections induced by tissue contraction are optically tracked.	Bending stiffness: 0.09–9.2 µN/µm	0.1–0.6 mN or 0.08–4 mN/mm^2^	Passive tension, beating rate, beating rhythm, contraction and relaxation time, Frank-Starling curve, force-velocity relationship	•Physiology: cardiomyocyte maturation •Drug testing: chromanol, quinidine, erythromycin, doxorubicin, isoproterenol, levosimendan, omecamtiv mecarbil •Disease modeling: cardiac hypertrophy and fibrosis, DCM	^50,54,132,156,158,190^
Biowires	3D tissue	NRVMs, iPSC-CMs, hESC-CMs	Wire bending induced by tissue contraction is optically tracked.	Diameter: 100 µm Young’s modulus: 33–250 kPa	1–40 µN	Passive tension, active force, beating rate, contraction and relaxation kinetics, maximum capture rate (electrical stimulation)	•Physiology: chamber-specific tissue response; cardiomyocyte maturation •Drug testing: isoproterenol, diltiazem, lidocaine, E-4031, verapamil, dofetilide, nifedipine, thapsigargin, milrinone •Disease modeling: HCM, DCM, fibrosis	^51,159,162,199^
Cardiac spheroids	3D tissue	NRVMs, iPSC-CMs, hESC-CMs	Video analysis (edge detection) is used to detect the spheroid fractional area change (indirect), or atomic force microscopy is performed (direct).	Video analysis: imaging frequency: 240 Hz AFM cantilever: spring constant: 0.025 N/m Sensitivity: 10.1 nm/V	Fractional area change: 0.2–1% AFM: 4.6–22 nN	Beating rate, average speed of contraction	•Drug testing: isoproterenol, metoprolol, verapamil, blebbistatin, ryanodine, caffeine, doxorubicin •Disease modeling: infarction, fibrosis	^38,166,170,172,173,200^
Engineered heart chamber	3D tissue	iPSC-CMs, NRVMs	Lumen pressure is detected with a catheter sensor. Stroke volume is detected with a high-speed video camera.	Depends on the specifications of camera and catheter sensor	1.26 mmH_2_O or 50 µmHg	Stroke volume, stroke work, ejection fraction, cardiac output, beating rate, pressure-volume relationship, Frank-Starling relationship	•Drug testing: isoproterenol, digoxin, verapamil, nifedipine, disopyramide, E-4031. •Disease modeling: structural arrhythmia	^136,174,175^

*CMs* cardiomyocytes, *NRVMs* neonatal rat ventricular myocytes, *hESC-CMs* human embryonic stem cell-derived CMs, *iPSC-CMs* induced pluripotent stem cell-derived CMs, *HCM* hypertrophic cardiomyopathy, *DCM* dilated cardiomyopathy, *MYBPC3* cardiac myosin-binding protein C3.

### Disease modeling

The incorporation of diseased iPSC-CMs onto contraction sensing platforms facilitates the investigation of disease mechanisms and the development of precision medicine. The disease-specific cardiomyocytes used for in vitro modeling are derived from patients with inherited cardiac diseases,^186,189^ produced through mutation via gene-editing techniques to recapitulate specific disease phenotypes^190^, or result from acquired dysfunction via maladaptive modeling due to pathogenic microenvironments^32,191^.

Hypertrophic cardiomyopathy (HCM) is one of the most common hereditary cardiac diseases, with ~60% of newly diagnosed patients possessing a family history^192^. HCM is characterized by abnormal thickening and stiffening of heart muscles that is typically caused by mutations in genes that encode thick and thin myofilaments and the Z-disc (e.g., MYH7, MYBPC3, TNNT2, and TNNI3 genes)^192^. Healy et al. utilized their filamentous matrix platform and iPSC-CMs deficient in the sarcomeric protein cardiac myosin-binding protein C (MYBPC3^−/−^) to establish a human HCM tissue model^162^. MYBPC3^−/−^ microtissues displayed reduced contraction only on matrices with relatively stiff fibers, suggesting that MYBPC3 deficiency and the presence of environmental stress (e.g., fibrosis) synergistically led to contractile deficits in the diseased cardiac tissue. Patients with dilated cardiomyopathy (DCM) show progressive left ventricular dilation, systolic dysfunction, and heart failure^193^. To investigate the pathogenic mechanism of titin-truncating variants (TTNtvs) in DCM, Hinson et al. established a microcantilever-based DCM tissue model cultured with patient-derived and CRISPR/CAS9-edited iPSC-CMs (Fig. 5c)^190^. The results demonstrated that the mutant titin protein caused a marked reduction in contractility and a loss of sarcomeres. Mitochondrial cardiomyopathy induced by Barth syndrome (caused by mutations in the *TAFAZZIN* gene) has been modeled on a flexible cantilever platform, demonstrating the application of the cantilever platform to quantify adverse tissue contraction changes that occur in mitochondrial genetic cardiac diseases in vitro^194^.

Acquired cardiac diseases have also been modeled on microengineered platforms via addition of pathological stimuli to in vitro cardiac models. Myocardial infarction (MI) caused by coronary artery blockage leads to decreased blood supply to the myocardium with permanent tissue damage^195^. Utilizing an oxygen-diffusion gradient in cardiac spheroids, an MI model was established under a hypoxic (10% oxygen) environment to recapitulate the organotypic features of post-MI cardiac injury^140^. Diseased spheroids with a gradient of apoptotic center>dysfunctional middle>functional edge reconstruct the anatomical concept of infarcted tissue>border zone>peri-infarct area-at-risk in the infarcted heart^196^. The diseased organoids exhibit significantly reduced contractile amplitudes and pathological metabolic shifts. After the occurrence of MI, clinical intervention to restore arterial blood flow to the infarcted area may induce further cardiomyocyte death and tissue injury (ischemia–reperfusion injury, IRI)^197^. Yadid et al. proposed an in vitro IRI model based on the cantilever platform to investigate the cardioprotective effects of endothelial extracellular vesicles (EEVs) on cardiomyocyte monolayers^191^. They found that EEVs efficiently attenuated cardiomyocyte death and loss of contractility during and after the IRI process. Cardiac fibrosis, also known as myocardial scarring, is a representative disease phenotype that occurs during the cardiac remodeling process after heart damage (e.g., MI) or under pathological stimuli (e.g., pressure overload)^198^. In vitro cardiac fibrosis tissue models have been established on the flexible wire system by either increasing the fibroblast percentage^199^ or by introducing transforming growth factor-β (TGF-β) to convert fibroblasts into diseased myofibroblasts^141^. Key features of the fibrotic myocardium, including fibroblast activation, excess deposition of ECM, tissue stiffening, and impaired contractility, have been modeled for antifibrotic drug testing. Recently, 3D bioprinting has been used to transfer cardiac spheroids into self-healing support hydrogels (Fig. 5d)^200^. The patterned healthy and scarred cardiac spheroids enable fusion into high-cell density microtissues for the creation of heterogeneous spatial tissue organization. The incorporation of contractility measurement with information from genetics, metabolomics, and proteomics has facilitated the investigation of cellular mechanisms underlying disease pathology.

## Summary and outlook

The advent of pluripotent stem cell technologies and heart-on-a-chip platforms has paved the way for emulation of human cardiac physiology and pathology in cell culture. The contraction force of cardiomyocytes is a key parameter reflecting normal or diseased cardiac function and is needed to evaluate the effects of pharmacological interventions. In this review, we described a variety of emerging approaches that have been developed to evaluate the contractile functions of in vitro cardiac models at the cellular and tissue levels. Platform designs, fabrication methods, sensing principles, cell types and model configurations have been described throughout this review. Table 1 summarizes existing platforms for quantifying the contractility of in vitro cardiac models through direct measurement of contractile stress, contractile force, and chamber pressure or through indirect measurement of beating velocity, beating displacement, beating strain, sarcomere shortening and impedance changes. In addition, beating parameters in the time scale, such as the beating rate, beating rhythm, and contraction and relaxation durations, can be extracted from the contraction signals to evaluate cardiac beating pace and regularity. The reported signal shape, magnitude range and polarities vary significantly among different sensing approaches and among different works using the same approach. Potential reasons include the differences in sensing principles, cell sources, differentiation protocols of induced cardiomyocytes, culture conditions, and model configurations. Although the extracted beating parameters in the time scale are comparable among different sensing methods, the lack of standardization in contractility measurement makes it difficult to directly compare the physical values of contractility magnitude reported in the literature. However, evaluating the relative changes in cardiac contractility is helpful to analyze and compare the responses of cardiomyocytes induced by microenvironmental stimulation, drug interventions, or cardiac disease.

In terms of platform preparation, some contraction sensing techniques only need commercial equipment and culture dishes, such as carbon fiber-based platforms^71^, AFM^80^, video-based motion detection equipment^42^, and cardiac spheroid culture dishes^38^. Other platforms are mostly fabricated by standard microfabrication technologies, including photolithography and silicon etching for the fabrication of MEMS force transducers^76^; metal deposition and patterning for the fabrication of impedance sensing electrodes and microcrack strain sensors^108,126^; hot embossing for the fabrication of Biowire platforms^51^; microcontact printing of ECM proteins for cell alignment^121^; and micromolding of soft polymer/hydrogel structures for the fabrication of micropillar arrays^53^, thin-film cantilevers^121^ and tissue posts^50^; and creation of the gel substrates required by TFM^42^. In addition, 3D printing and micromilling are applied for rapid prototyping^52,141^. The utilization of standard microfabrication technologies ensures batch fabrication of micro-/nanostructures with high accuracy and repeatability. Efforts in platform development will focus on simplifying the fabrication process, improving sensing performance, reducing cost and scaling up for high-throughput testing.

With regard to sensing principles, optical-based measurements are the main methods for contraction analysis through tracking of the cell/tissue shape change or the deformation of attached flexible structures^51,100,166,201,202^. However, the relationship between cell/tissue shape changes and their produced contractile force is challenging to correlate, especially when the changes need to be translated into cellular physiology. Other factors, such as cell/tissue stiffness, also influence the magnitude of the fractional shape change. Optical-based methods require taking cells/tissues out of the incubator for imaging under a microscope, which may disturb their beating behaviors. Integrating electrical biosensors into in vitro cardiac models enables in situ and continuous characterization of contractile function. IDE biosensors have been used to achieve continuous measurement of cardiomyocyte contraction by detecting the contraction-induced impedance signal changes^203,204^. In addition, advances in flexible electronics and sensing materials have provided new opportunities to integrate flexible sensors onto heart-on-a-chip platforms for contractility measurement inside the incubator environment^52,120,126,205^. Strain sensors based on thin metal films^49,125^, highly stretchable strain sensors based on CNTs^120^ and carbon black composites^52^, and high-sensitivity crack sensors^126^ have been integrated with suspended membrane substrates and thin-film cantilever platforms, which exhibit excellent sensing performance for long-term and continuous detection of the contractile behaviors of 2D monolayers of cardiomyocytes. In comparison, contraction measurements of 3D cardiac tissues currently rely on optical imaging^51,166,170^. Direct electrical readout of contractile functions from 3D cardiac tissue models has yet to be realized.

Cardiomyocyte contraction follows excitation-contraction coupling, a complex cascade of ion channel activities, cytosolic calcium fluxes and the shortening of contractile filaments^44^. Hence, simultaneous detection of multiple functional parameters is necessary to provide comprehensive insight into the mechanisms of contraction changes under physiological conditions or drug stimulation. Previous works on multiparameter measurements have used different techniques and platforms and collected information from individual experimental tests^129,154,206^. Separate testing of different functional parameters increases the experimental workload and, more importantly, misses critical information such as the time-dependent relationship among contractility, electrophysiology, and calcium dynamics. Thus, there is a trend toward integrating different biosensing components onto a single platform to simultaneously perform multiparameter measurements in a cell/tissue culture environment. Multiparameter analysis of cardiac functional properties in vitro has recently been reported^68,104,108,207^. For example, a label-free electromechanical detection strategy based on microelectrodes and IDEs can synchronously monitor the electrophysiology and contraction of 2D cardiomyocyte monolayers^56,208^. Van Meer et al. developed a high-speed optical system capable of measuring contraction, action potential, and calcium influx simultaneously using fluorescent voltage- and calcium-sensitive dyes and live membrane-labeling dyes^104^. Simultaneous fluorescence imaging was achieved by high-speed filter switching in the emission pathway. Multiplexing of these three functional features of iPSC-CMs was achieved; however, the potential cytotoxicity introduced by the fluorescent dyes may impede long-term and accurate monitoring of cardiac functional parameters.

Drug testing and disease modeling in a single-cardiomyocyte model show advantages for analyzing the drug/disease-induced contractile responses of adult cardiomyocytes isolated from animal or human hearts, which could alleviate potential bias induced by the immature phenotypes of neonatal or stem cell-induced cardiomyocytes^209,210^. However, testing inotropic effects at the single-cell level is complex to implement, with low assay throughput and difficulties in detecting chronic drug effects or anti-arrhythmic drugs targeting gap junctions^106^. 2D cardiomyocyte monolayer models have been widely utilized by pharmaceutical companies to evaluate drug therapeutic effects or cardiotoxicity^18,118,181^. The advantages of the 2D monolayer model include simple culture procedures, a low cost, and recapitulation of synchronous cardiac beating and intercellular communication. Recently, developed multiwall contraction sensing platforms have greatly promoted the efficiency of batch screening of the inotropic, chronotropic and lusitropic effects of preclinical drugs^120,183^. However, cardiomyogenesis in the 2D formation differs from that in the 3D tissue formation in terms of cell morphology, polarity, intercellular adhesion structures, mitochondrial morphology, ECM distribution, and protein expression^109^. Engineered cardiac tissue models have demonstrated advantages in disease modeling and drug testing: they mimic 3D tissue morphologies, cell compositions and ECM microenvironments. In addition to cardiomyocytes, several types of supporting cells, such as cardiac fibroblasts, smooth muscle cells, endothelial cells and immune cells, collectively occupy a small volume fraction (~25–30%) of the native myocardium but play critical roles in heart homeostasis maintenance, heart repair, ECM production and nutrient supply^66,211,212^. The benefit of coculturing cardiomyocytes with supporting cells in 3D cardiac tissue models is that it can recapitulate the interactions between different cell types either through cell–cell contact or paracrine factors, which has been proven to strongly influence the function of the myocardium^145^. For example, tissue models have been used to model cardiac damage or myocardial fibrosis at the tissue level and to screen drug candidates for tissue stiffness reduction, myofibroblast activation and ECM deposition^141,199^. However, current 3D cardiac tissue models still require complex tissue culture protocols and platforms^134,161^, with significant monetary and time costs. Standardizing culture processes, improving tissue reproducibility, and efficiently characterizing tissue functional properties demand further research efforts. Furthermore, heart-on-a-chip models can also be functionally incorporated into multiple-organs-on-a-chip systems to reconstitute body metabolism and physiology for analysis of multiorgan interactions, having the potential to serve as a replacement for animal models to evaluate synergistic responses to pharmaceutical compounds (e.g., drug accumulation, distribution, and cardiotoxicity)^213,214^.

It needs to be noted that cell physiologies and microenvironments in vivo are more complex than in vitro representations. Since the biophysical and biochemical microenvironments play a critical role in directing heart development, maintaining homeostasis, and regulating the pathogenetic process^180,215^, it is necessary to incorporate controlled microenvironmental stimuli together with biosensing technologies to investigate cardiac cell/tissue remodeling processes under physiological and pathophysiological conditions. One concern is that the majority of iPSC-CMs remain immature in structure and function compared with primary cardiomyocytes and are therefore associated with high intersample variability^27^.

To mitigate this concern, integration of microenvironmental stimuli on the heart-on-a-chip platform has been utilized to drive iPSC-CMs toward more adult-like phenotypes. For example, application of mechanical loading has been proven to upregulate the contractility, sarcomere elongation and relevant gene expression of both 2D iPSC-CM monolayers and 3D cardiac tissues in a loading magnitude-dependent manner^42,50^. The enhancement of contractility plateaus at a moderate mechanical loading magnitude (~15%), while excess mechanical loading can increase the gene expression of pathological biomarkers related to cardiac hypertrophy and fibrosis^50,130^. TFM has been applied to investigate the influence of substrate stiffness on the functional maturation of single cardiomyocytes and 2D monolayers^98,216^. Gel substrates with stiffness comparable to the native myocardial stiffness (10–20 kPa) have been found to optimize cell responses in contractile force development, calcium transients, sarcomere alignment and cell striations. In addition to mechanical cues, electrical stimulation has been applied to induce maturation of in vitro cardiac models^154,217^. Increasing the electrical stimulation frequency during cell culture has been shown to induce greater enhancement of contractility, calcium handling, sarcomere structures and cell alignment than fixed-rate electrical pacing on the Biowire platform^154,187^. In particular, applying ramped electrical stimulation to 3D cardiac tissues generated from the early differentiation stage of iPSC-CMs results in remarkable tissue maturation progress with sarcomere lengths, T-tubules, mitochondrial density, action potential curves and mRNA expression comparable to those of the human adult myocardium, but deficient contractile forces are still measured by flexible pillars^40^. In terms of biochemical cues, Hofbauer et al. recently established a self-organizing cardioid derived from induced stem cells and proved the importance of the Wnt-BMP signaling axis in the formation of chamber-like structures and cardiac contractile functions during human cardiogenesis^218^. Flexible tissue wire platforms have also been utilized to investigate the degradation of contractility in diseased tissue models triggered by biochemical factors, including TGF-β-induced cardiac fibrosis^141^ and angiotensin II-induced progressive cardiomyopathy^160^. Future studies are expected to further screen and elucidate the integrative roles of different microenvironmental cues in directing cell development, functional properties, and pathogenetic processes.

Building upon these strong foundations, the next decade will see continued advances in both technology (e.g., fabrication techniques, sensing materials, and platform designs) and biology (e.g., maturation of cardiomyocytes and optimization of culturing protocols) to achieve continuous, multiparameter and high-throughput functional measurement of cardiac models that more comprehensively recapitulate native in vivo conditions and reflect holistic interactions among different cell and organ types. This will further spur investigations into disease mechanisms and the development of more precise and efficacious treatment paradigms.
